# 1113. Oral Tebipenem as Step-Down Therapy Following Intravenous Ertapenem in a 7-day Hollow-Fiber *In Vitro* Infection Model

**DOI:** 10.1093/ofid/ofab466.1307

**Published:** 2021-12-04

**Authors:** Brian D VanScoy, Sean Jones, Haley Conde, Lawrence Friedrich, Nicole Cotroneo, Paul G Ambrose

**Affiliations:** 1 Institute for Clinical Pharmacodynamics, Inc., Schenectady, New York; 2 Institute for Clinical Pharmacodynamics, Schenectady, New York; 3 Spero Therapeutics, Cambridge, Massachusetts

## Abstract

**Background:**

Intravenous (IV) to oral (PO) antibiotic step-down therapy has many benefits including reducing the length of hospital stay and lowering the risk of nosocomial infections and overall cost. While fluoroquinolones have been utilized as a step-down therapy for urinary tract infections (UTI) for some time, increases in fluoroquinolone-resistant *Escherichia coli* makes this an increasingly unsuitable option. Tebipenem (TBP) is an orally bioavailable carbapenem administered as a pro-drug (tebipenem pivoxil hydrobromide) with broad-spectrum activity that is currently in development for the treatment of patients with complicated UTI. Herein we describe a series of 7-day hollow-fiber *in vitro* infection model (HFIM) assays designed to evaluate the potential of PO TBP as step-down therapy from IV ertapenem (ETP).

**Methods:**

A panel of ESBL+ clinical *E. coli* isolates, selected based upon ETP and TBP MIC values and known resistance profiles, were evaluated at an initial burden of 10^7^ CFU/mL. HFIM, ETP and TBP free-drug concentration-time profiles were simulated in the HFIM to represent those following IV and PO dosing, respectively. Each isolate was challenged in duplicate by ETP administered every 24 hours (q24h) for 1, 3 or 7 days of therapy. TBP 600 mg every 8 hours was simulated as a step-down therapy following either 1 or 3 days of ETP treatment as well as a 7-day monotherapy regimen. All active regimens were compared to a no-treatment control as well as ETP 1g q24h for 1 or 3 days followed by a halting of therapy. Samples were collected for enumeration of bacterial populations and observation of simulated PK profiles throughout.

**Results:**

Bacteria grew well in the no-treatment control group, reaching densities > 10^10^ CFU/mL. ETP 1g q24h monotherapy successfully reduced bacterial burdens to below levels of detection over the 7-day period but was followed by regrowth matching that in the no-treatment control when treatment was halted on Days 1 and 3. Similar to ETP, TBP successfully reduced bacterial burdens as a 7-day monotherapy and following 1 and 3 days of IV ETP by preventing growth throughout the remainder of the study.

**Conclusion:**

These data demonstrate the potential utility of TBP as oral step-down from IV ETP therapy and the need for evaluation as a step-down from other IV therapeutics.

**Disclosures:**

**Brian D. VanScoy, B.S.**, **3-V Biosciences** (Grant/Research Support)**Achogen** (Grant/Research Support)**Amplyx Pharmaceuticals, Inc.** (Grant/Research Support)**Arixa Pharmaceuticals** (Grant/Research Support)**Arsanis Inc.** (Grant/Research Support)**B. Braun Medical Inc.** (Grant/Research Support)**Basilea Pharmaceutica** (Grant/Research Support)**BLC USA** (Grant/Research Support)**Boston Pharmaceuticals** (Grant/Research Support)**Bravos Biosciences, LLC** (Grant/Research Support)**Cidara Therapeutics Inc.** (Grant/Research Support)**Cipla, USA** (Grant/Research Support)**Corcept Therapeutics** (Grant/Research Support)**Cumberland Pharmaceuticals** (Grant/Research Support)**Debiopharm International SA** (Grant/Research Support)**Discuva Limited** (Grant/Research Support)**Emerald Lake Technologies** (Grant/Research Support)**Enhanced Pharmacodynamics** (Grant/Research Support)**Entasis Therapeutics** (Grant/Research Support)**E-Scape Bio** (Grant/Research Support)**Genentech** (Grant/Research Support)**Geom Therapeutics, Inc.** (Grant/Research Support)**GlaxoSmithKline** (Grant/Research Support)**Hoffmann-La Roche** (Grant/Research Support)**Horizon Orphan LLC** (Grant/Research Support)**ICPD Biosciences, LLC** (Grant/Research Support)**Indalo Therapeutics** (Grant/Research Support)**Insmed Inc.** (Grant/Research Support)**Institute for Clinical Pharmacodynamics** (Employee)**Iterum** (Grant/Research Support)**KBP Biosciences USA** (Grant/Research Support)**Kyoto Biopharma, Inc.** (Grant/Research Support)**Matinas** (Grant/Research Support)**Meiji Seika Pharma Co., Ltd.** (Grant/Research Support)**Melinta Therapeutics** (Grant/Research Support)**Menarini Ricerche S.p.A.** (Grant/Research Support)**Merck & Co., Inc** (Grant/Research Support)**Mutabilis** (Grant/Research Support)**Nabriva Therapeutics AG** (Grant/Research Support)**Naeja-RGM Pharmaceuticals** (Grant/Research Support)**Nosopharm SAS** (Grant/Research Support)**Novartis Pharmaceuticals Corp.** (Grant/Research Support)**NuCana Biomed** (Grant/Research Support)**Paratek Pharmaceuticals, Inc.** (Grant/Research Support)**Polyphor, Ltd.** (Grant/Research Support)**Prothena Corporation** (Grant/Research Support)**PTC Therapeutics** (Grant/Research Support)**Rempex Pharmaceuticals** (Grant/Research Support)**Roche TCRC** (Grant/Research Support)**Sagimet** (Grant/Research Support)**scPharmaceuticals Inc.** (Grant/Research Support)**Scynexis** (Grant/Research Support)**Spero Therapeutics** (Grant/Research Support)**TauRx Therapeutics** (Grant/Research Support)**Tetraphase Pharmaceuticals** (Grant/Research Support)**Theravance Biopharma Pharmaceutica** (Grant/Research Support)**USCAST** (Grant/Research Support)**VenatoRx** (Grant/Research Support)**Vical Incorporated** (Grant/Research Support)**Wockhardt Bio AG** (Grant/Research Support)**Zavante Therapeutics** (Grant/Research Support)**Zogenix International** (Grant/Research Support) **Sean Jones, B.S.**, **3-V Biosciences** (Grant/Research Support)**Achogen** (Grant/Research Support)**Amplyx Pharmaceuticals, Inc.** (Grant/Research Support)**Arixa Pharmaceuticals** (Grant/Research Support)**Arsanis Inc.** (Grant/Research Support)**B. Braun Medical Inc.** (Grant/Research Support)**Basilea Pharmaceutica** (Grant/Research Support)**BLC USA** (Grant/Research Support)**Boston Pharmaceuticals** (Grant/Research Support)**Bravos Biosciences, LLC** (Grant/Research Support)**Cidara Therapeutics Inc.** (Grant/Research Support)**Cipla, USA** (Grant/Research Support)**Corcept Therapeutics** (Grant/Research Support)**Cumberland Pharmaceuticals** (Grant/Research Support)**Debiopharm International SA** (Grant/Research Support)**Discuva Limited** (Grant/Research Support)**Emerald Lake Technologies** (Grant/Research Support)**Enhanced Pharmacodynamics** (Grant/Research Support)**Entasis Therapeutics** (Grant/Research Support)**E-Scape Bio** (Grant/Research Support)**Genentech** (Grant/Research Support)**Geom Therapeutics, Inc.** (Grant/Research Support)**GlaxoSmithKline** (Grant/Research Support)**Hoffmann-La Roche** (Grant/Research Support)**Horizon Orphan LLC** (Grant/Research Support)**ICPD Biosciences, LLC** (Grant/Research Support)**Indalo Therapeutics** (Grant/Research Support)**Insmed Inc.** (Grant/Research Support)**Institute for Clinical Pharmacodynamics** (Employee)**Iterum** (Grant/Research Support)**KBP Biosciences USA** (Grant/Research Support)**Kyoto Biopharma, Inc.** (Grant/Research Support)**Matinas** (Grant/Research Support)**Meiji Seika Pharma Co., Ltd.** (Grant/Research Support)**Melinta Therapeutics** (Grant/Research Support)**Menarini Ricerche S.p.A.** (Grant/Research Support)**Merck & Co., Inc** (Grant/Research Support)**Mutabilis** (Grant/Research Support)**Nabriva Therapeutics AG** (Grant/Research Support)**Naeja-RGM Pharmaceuticals** (Grant/Research Support)**Nosopharm SAS** (Grant/Research Support)**Novartis Pharmaceuticals Corp.** (Grant/Research Support)**NuCana Biomed** (Grant/Research Support)**Paratek Pharmaceuticals, Inc.** (Grant/Research Support)**Polyphor, Ltd.** (Grant/Research Support)**Prothena Corporation** (Grant/Research Support)**PTC Therapeutics** (Grant/Research Support)**Rempex Pharmaceuticals** (Grant/Research Support)**Roche TCRC** (Grant/Research Support)**Sagimet** (Grant/Research Support)**scPharmaceuticals Inc.** (Grant/Research Support)**Scynexis** (Grant/Research Support)**Spero Therapeutics** (Grant/Research Support)**TauRx Therapeutics** (Grant/Research Support)**Tetraphase Pharmaceuticals** (Grant/Research Support)**Theravance Biopharma Pharmaceutica** (Grant/Research Support)**USCAST** (Grant/Research Support)**VenatoRx** (Grant/Research Support)**Vical Incorporated** (Grant/Research Support)**Wockhardt Bio AG** (Grant/Research Support)**Zavante Therapeutics** (Grant/Research Support)**Zogenix International** (Grant/Research Support) **Haley Conde, B.S.**, **3-V Biosciences** (Grant/Research Support)**Achogen** (Grant/Research Support)**Amplyx Pharmaceuticals, Inc.** (Grant/Research Support)**Arixa Pharmaceuticals** (Grant/Research Support)**Arsanis Inc.** (Grant/Research Support)**B. Braun Medical Inc.** (Grant/Research Support)**Basilea Pharmaceutica** (Grant/Research Support)**BLC USA** (Grant/Research Support)**Boston Pharmaceuticals** (Grant/Research Support)**Bravos Biosciences, LLC** (Grant/Research Support)**Cidara Therapeutics Inc.** (Grant/Research Support)**Cipla, USA** (Grant/Research Support)**Corcept Therapeutics** (Grant/Research Support)**Cumberland Pharmaceuticals** (Grant/Research Support)**Debiopharm International SA** (Grant/Research Support)**Discuva Limited** (Grant/Research Support)**Emerald Lake Technologies** (Grant/Research Support)**Enhanced Pharmacodynamics** (Grant/Research Support)**Entasis Therapeutics** (Grant/Research Support)**E-Scape Bio** (Grant/Research Support)**Genentech** (Grant/Research Support)**Geom Therapeutics, Inc.** (Grant/Research Support)**GlaxoSmithKline** (Grant/Research Support)**Hoffmann-La Roche** (Grant/Research Support)**Horizon Orphan LLC** (Grant/Research Support)**ICPD Biosciences, LLC** (Grant/Research Support)**Indalo Therapeutics** (Grant/Research Support)**Insmed Inc.** (Grant/Research Support)**Institute for Clinical Pharmacodynamics** (Employee)**Iterum** (Grant/Research Support)**KBP Biosciences USA** (Grant/Research Support)**Kyoto Biopharma, Inc.** (Grant/Research Support)**Matinas** (Grant/Research Support)**Meiji Seika Pharma Co., Ltd.** (Grant/Research Support)**Melinta Therapeutics** (Grant/Research Support)**Menarini Ricerche S.p.A.** (Grant/Research Support)**Merck & Co., Inc** (Grant/Research Support)**Mutabilis** (Grant/Research Support)**Nabriva Therapeutics AG** (Grant/Research Support)**Naeja-RGM Pharmaceuticals** (Grant/Research Support)**Nosopharm SAS** (Grant/Research Support)**Novartis Pharmaceuticals Corp.** (Grant/Research Support)**NuCana Biomed** (Grant/Research Support)**Paratek Pharmaceuticals, Inc.** (Grant/Research Support)**Polyphor, Ltd.** (Grant/Research Support)**Prothena Corporation** (Grant/Research Support)**PTC Therapeutics** (Grant/Research Support)**Rempex Pharmaceuticals** (Grant/Research Support)**Roche TCRC** (Grant/Research Support)**Sagimet** (Grant/Research Support)**scPharmaceuticals Inc.** (Grant/Research Support)**Scynexis** (Grant/Research Support)**Spero Therapeutics** (Grant/Research Support)**TauRx Therapeutics** (Grant/Research Support)**Tetraphase Pharmaceuticals** (Grant/Research Support)**Theravance Biopharma Pharmaceutica** (Grant/Research Support)**USCAST** (Grant/Research Support)**VenatoRx** (Grant/Research Support)**Vical Incorporated** (Grant/Research Support)**Wockhardt Bio AG** (Grant/Research Support)**Zavante Therapeutics** (Grant/Research Support)**Zogenix International** (Grant/Research Support) **Lawrence Friedrich, Pharm.D.**, **Spero Therapeutics** (Employee, Shareholder) **Nicole Cotroneo**, **Spero Therapeutics** (Employee, Shareholder) **Paul G. Ambrose, Pharm.D., FIDSA**, **3-V Biosciences** (Grant/Research Support)**Achogen** (Grant/Research Support)**Amplyx Pharmaceuticals, Inc.** (Grant/Research Support)**Arixa Pharmaceuticals** (Grant/Research Support)**Arsanis Inc.** (Grant/Research Support)**B. Braun Medical Inc.** (Grant/Research Support)**Basilea Pharmaceutica** (Grant/Research Support)**BLC USA** (Grant/Research Support)**Boston Pharmaceuticals** (Grant/Research Support)**Bravos Biosciences, LLC** (Grant/Research Support, Other Financial or Material Support, member/owner)**Cidara Therapeutics Inc.** (Grant/Research Support)**Cipla, USA** (Grant/Research Support)**Corcept Therapeutics** (Grant/Research Support)**Cumberland Pharmaceuticals** (Grant/Research Support)**Debiopharm International SA** (Grant/Research Support)**Discuva Limited** (Research Grant or Support)**Emerald Lake Technologies** (Grant/Research Support)**Enhanced Pharmacodynamics** (Grant/Research Support)**Entasis Therapeutics** (Grant/Research Support)**E-Scape Bio** (Grant/Research Support)**Genentech** (Grant/Research Support)**Geom Therapeutics, Inc.** (Grant/Research Support)**GlaxoSmithKline** (Grant/Research Support)**Hoffmann-La Roche** (Grant/Research Support)**Horizon Orphan LLC** (Grant/Research Support)**ICPD Biosciences, LLC** (Grant/Research Support, Other Financial or Material Support, member/owner)**Indalo Therapeutics** (Grant/Research Support)**Insmed Inc.** (Grant/Research Support)**Institute for Clinical Pharmacodynamics** (Employee)**Iterum** (Grant/Research Support)**KBP Biosciences USA** (Grant/Research Support)**Kyoto Biopharma, Inc.** (Grant/Research Support)**Matinas** (Grant/Research Support)**Meiji Seika Pharma Co., Ltd.** (Grant/Research Support)**Melinta Therapeutics** (Grant/Research Support)**Menarini Ricerche S.p.A.** (Grant/Research Support)**Merck & Co., Inc** (Grant/Research Support)**Mutabilis** (Grant/Research Support)**Nabriva Therapeutics AG** (Grant/Research Support)**Naeja-RGM Pharmaceuticals** (Grant/Research Support)**Nosopharm SAS** (Grant/Research Support)**Novartis Pharmaceuticals Corp.** (Grant/Research Support)**NuCana Biomed** (Grant/Research Support)**Paratek Pharmaceuticals, Inc.** (Grant/Research Support)**Polyphor, Ltd.** (Grant/Research Support)**Prothena Corporation** (Grant/Research Support)**PTC Therapeutics** (Grant/Research Support)**Rempex Pharmaceuticals** (Grant/Research Support)**Roche TCRC** (Grant/Research Support)**Sagimet** (Grant/Research Support)**scPharmaceuticals Inc.** (Grant/Research Support)**Scynexis** (Grant/Research Support)**Spero Therapeutics** (Grant/Research Support)**TauRx Therapeutics** (Grant/Research Support)**Tetraphase Pharmaceuticals** (Grant/Research Support)**Theravance Biopharma Pharmaceutica** (Grant/Research Support)**USCAST** (Grant/Research Support)**VenatoRx** (Grant/Research Support)**Vical Incorporated** (Grant/Research Support)**Wockhardt Bio AG** (Grant/Research Support)**Zavante Therapeutics** (Grant/Research Support)**Zogenix International** (Grant/Research Support)

